# Suppression of metastasis of intratesticular inoculation of B16FO melanoma cells by a novel nutrient mixture in male athymic nude mice

**DOI:** 10.3892/etm.2012.689

**Published:** 2012-08-31

**Authors:** M. WAHEED ROOMI, TATIANA KALINOVSKY, NUSRATH W. ROOMI, ALEKSANDRA NIEDZWIECKI, MATTHIAS RATH

**Affiliations:** Dr Rath Research Institute, Oncology Division, 1260 Memorex Drive, Santa Clara, CA, USA

**Keywords:** metastasis, intratesticular injection, melanoma B16FO cells, athymic nude mice, nutrient mixture

## Abstract

Metastasis, commonly to the lung, is the major cause of mortality from testicular cancer. The aim of the present study was to examine the effect of a novel nutrient mixture (NM) containing ascorbic acid, amino acids and green tea extract on the inhibition of melanoma growth and metastasis using a model of intratesticular inoculation of B16FO cells into nude mice. Male athymic mice (n=12), 10–12 weeks of age, were inoculated with 5×10^5^ B16FO melanoma cells in 100 μl of PBS into the right testis, while the left testis was left untreated. Following inoculation, the mice were randomly divided into two groups. The control group (n=6) was fed a regular mouse chow diet and the NM 1% group (n=6) the same diet, but supplemented with 1% NM. Four weeks later the mice were sacrificed and the abdominal cavity was opened. Mice in the control group exhibited extensive metastasis in the peritoneal cavity and severely enlarged right testes and necrotic seminiferous tubules. By contrast, in the NM 1% fed group there was no evidence of peritoneal metastasis in 50% of the animals and mild metastasis in the remaining 50%. The right testes were enlarged and seminiferous tubules in the area of invasion showed evidence of degeneration. No metastasis to the liver, kidney or spleen were evident in either group. However, severe lung metastasis was observed in 2 of 6 mice in the control group and mild metastasis in 2 of 6 mice in the NM 1% group. In conclusion, these results confirm earlier studies and verify the anti-metastatic potential of NM.

## Introduction

Testicular cancer (TC) is a relatively rare type of cancer that may often leads to metastasis. Approximately 8,590 new cases are likely to be diagnosed in the United States in 2012, of which 360 individuals are expected to succumb to the disease (1). Although the disease is not common, as the likelihood of developing testicular cancer is approximately 1 in 270, the rate of testicular cancer has been on the increase in the United States and many other countries, with the increase mostly in seminomas (1). Caucasians have 5 times the risk of developing the disease compared to African-Americans and 3 times that of Asian-Americans (1). As yet, no rationale for the increase has been found. TC affects males of all ages although 90% of cases occur in men between the ages of 20 and 54 years. Factors that increase the risk of developing testicular cancer include undescended testicle, Kleinfelder syndrome, family history of testicular cancer, HIV infection, particularly in those with AIDS, carcinoma *in situ*, and cancer of the other testicle (1). TC is considered to be one of the most curable forms of cancer. However, if the cancer has metastasized beyond the lymph nodes, the 5-year survival is reduced to 71% (1). The primary modality of spread of TC is through the lymph node system to the retroperitoneum and in certain cases to other lymph nodes along the mid-line of the body. Spread continues through the bloodstream, common in patients with advanced germ cell tumors or those with choriocarcinoma or embryonal carcinoma elements. The main sites for blood-borne metastatic tumors are the lungs, followed by the liver, bone and brain (2,3).

Metastatic malignant melanoma cells, specifically B16, have been successfully utilized for experimental metastasis to study the effectiveness of anticancer agents, since melanoma cells are extremely aggressive and metastasize to secondary areas of the body, such as lymph nodes, lungs, liver, brain or bone (4). Hart and Fidler (4) studied the role of organ selectivity in the determination of metastatic patterns of B16 melanoma and concluded that the outcome of metastasis was dependent on tumor cell properties and host factors, supporting the ‘seed and soil’ hypothesis to explain the non-random pattern of cancer metastasis. For example, although the circulatory mode of spread leads to the dissemination of a number of malignant cells, it cannot fully explain the patterns of distribution of numerous tumors, such as the infrequent metastatic development in organs including the spleen or skeletal muscle with highly developed vasculature. In their study, Zeidman and Busso (5) reported that tumor cells from different tumors interacted differently with the capillary bed of a given organ, while Sugarbaker *et al* (6) found that tumor cell suspensions from different types of tumors injected into the same site in rats established different patterns of metastases. Experimental data have indicated that melanoma B16 cells preferentially metastasize to specific organs, such as the lungs and liver (4,7,8).

A nutrient mixture (NM) containing lysine, proline, ascorbic acid and green tea extract has demonstrated anticancer activity in a number of human cancer cell lines, inhibiting cancer cell growth, MMP secretion, invasion, metastasis and angiogenesis (9). In a previous study, we demonstrated that NM was effective in inhibiting the pulmonary metastasis of B16FO melanoma cells injected into the tail vein of C57BL/6 mice, especially when nutrients were delivered intravenously or intraperitoneally (7). We also demonstrated the effectiveness of dietary supplementation with NM to prevent experimental hepatic metastasis by studying its effect on the intrasplenic injection of B16FO cells into athymic nude mice (10).

The aim of this study was to investigate the effect of NM on the experimental metastasis of melanoma cells by the intratesticular injection of B16FO cells into male nude mice. This experimental model of metastasis was selected to study the effectiveness of nutrients against metastasis to the lungs and other organs from the testes since melanoma cells are aggressive enough to result in significant metastasis in mice, particularly pulmonary metastasis, a common end organ of metastasis for testicular cancer. Furthermore, the intratesticular model was found to be an effective model for studying mechanisms of metastasis and evaluating treatment strategies due to the stable formation of tumors with metastatic potential (11).

## Materials and methods

### Cancer cell line and culture

Murine melanoma B16FO cells obtained from ATCC (American Type Culture Collection, Rockville, MD, USA) were maintained in Dulbecco’s modified Eagle’s medium (DMEM) supplemented with 10% fetal bovine serum, 100 U/ml penicillin and 100 μg/ml streptomycin. The media and sera used were obtained from ATCC, while the antibiotics (penicillin and streptomycin) were purchased from Gibco-BRL (Long Island, NY, USA).

### Animals

Male nude mice, approximately 9–11 weeks of age on arrival, were purchased from Simonsen Laboratories (Gilroy, CA, USA), and kept in microisolator cages under pathogen-free conditions on a 12-h light/dark schedule for a week. The animals were cared for in accordance with institutional guidelines for the care and use of experimental animals.

### Diet

The regular rodent diet was obtained from Purina Mills (Gray Summit, MO, USA). The NM 1% supplemented diet mix was milled and pressed by Purina Mills, LLC, and generated by Vitatech (Tustin, CA, USA). The NM 1% diet comprised the following in the ratio indicated: vitamin C (as ascorbic acid and as Mg, Ca, and palmitate ascorbate) 700 mg; L-lysine 1000 mg; L-proline 750 mg; L-arginine 500 mg; N-acetyl cysteine 200 mg; standardized green tea extract (80% polyphenol) 1000 mg; selenium 30 μg; copper 2 mg; manganese 1 mg.

### Experimental design

Male athymic mice (n=12), 10–12 weeks of age, were anesthetized by inhalation utilizing isofluorane USP (Abbott Laboratories, Chicago, IL, USA). The right side of the abdomen overlying the testis was sterilely prepped and a skin incision of 1 cm was made to expose the right testis. Mice were inoculated with 5×10^5^ B16FO melanoma cells in 100 μl of PBS into the right testis, while the left testis was left untreated. The cavities were sutured and clamped. After inoculation, the mice were randomly divided into 2 groups. The control group (n=6) was fed regular Purina mouse chow diet, while the mice in the NM 1% group (n=6) were fed the same diet, but supplemented with 1% NM. The quantity of diet provided to the mice was unrestricted, however, the mice consumed, on average, 4 g of their respective diets/day. Thus, the supplemented mice received ∼40 mg of NM/day, indicating that they received the following amounts of NM components/day: ascorbic acid 7 mg, L-lysine 10 mg, green tea extract 10 mg, L-proline 7.5 mg, L-arginine 5 mg, N-acetyl cysteine 2 mg, selenium 0.3 μg, copper 0.02 mg, manganese 10 μg. Four weeks later the mice were sacrificed, the abdominal cavity was opened and testes, lungs, kidneys, livers and spleens were excised from all the animals and examined. Since growth of testes in the control animals expanded profoundly into the peritoneum making it difficult to determine the limits of the organs, measurements of volume and weight were not carried out. Growth of melanoma colonies in testicles were evaluated by sectioned tissue. Lung metastasis was evaluated by observation of the melanoma colonies. A control mouse was sacrificed and examined at 1 week. All procedures were performed according to humane and customary care and use of experimental animals and conducted under protocols approved by the Internal Animal Care and Use Committee (IACUC).

### Histopathology

Testicular tissues were fixed in 10% buffered formalin, embedded in paraffin and cut at 4–5 microns. Sections were deparaffininzed through xylene and graduated alcohol series to water, and stained with hematoxylin and eosin (H&E) for microscopic evaluation by IDEXX Reference Laboratories.

## Results

### Melanoma growth in testes and peritoneal metastasis

**Figure 1:**
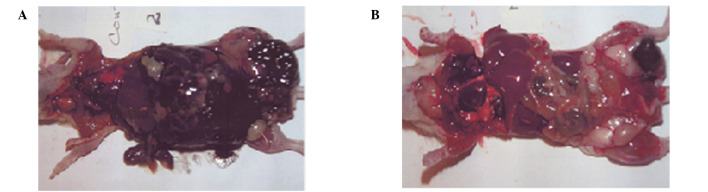
Representative gross images of peritoneal cavities of nude mice injected with B16FO melanoma cells fed the control or NM 1% diet (4 weeks post-injection) are shown. (A) Control diet group and (B) NM 1% diet group.

**Figure 2:**
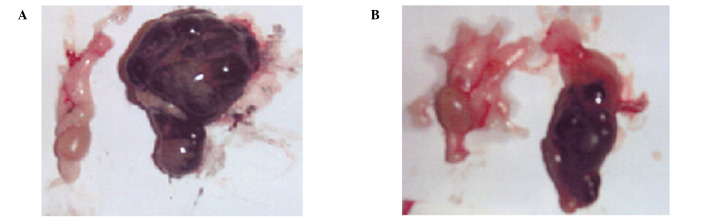
Representative gross images of nude mice testes in the control and NM 1% diet groups are shown. The left testis was untreated and the right testis was injected with B16FO melanoma cells (4 weeks post-injection). (A) Control diet group and (B) NM 1% diet group.

**Figure 3:**
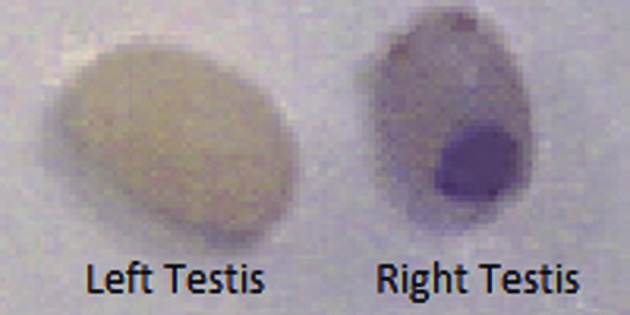
Representative gross photos and photomicrographs of testes from nude mice fed the dontrol diet (1 week post injection). Left testis (untreated), right testis (injected with B16FO cells).

The mice (6/6) in the control group exhibited extensive metastasis in the peritoneal cavity, which was totally masked by B16FO melanoma cells, in contrast to the NM 1% group, which showed no peritoneal metastasis in 3 mice and mild metastasis in 3 mice. Representative images of the peritoneum in the two groups are shown in Fig. 1. The right testis in the control group was severely enlarged and replaced by invading malignant melanoma cells and the remaining testicular tissue was represented by necrotic seminiferous tubules. The capsular region of the testis was severely infiltrated with a population of mixed cells. By contrast, in the NM 1% group, the testes were slightly enlarged and the seminiferous tubules in the area of invasion showed evidence of degeneration. The left testes of the two groups shows no evidence of melanoma colonies; however, the right testes of the control group of nude mice shows extensive melanoma invasion, while the NM 1% group shows mild invasion of melanoma cells (Fig. 2). Profound enlargement of the right testis was observed in the control group mice. By contrast, some enlargement of the right testis was evident in the NM 1% group, although it was much smaller than that observed in the control group. Representative gross images of the left and right testes in the control group at 1 week post-injection are shown in Fig. 3.

### Histopathology of representative testicular sections

**Figure 4:**
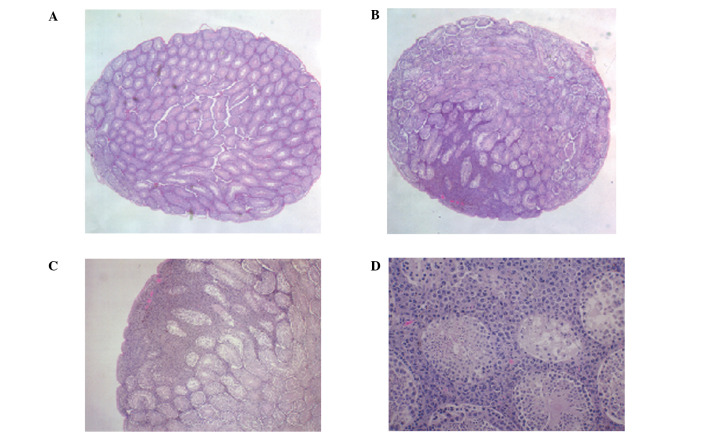
Representative photomicrographs of untreated testis and B16FO-injected testis in the control group (1 week post-injection). (A) Left (untreated) testis, (B) right (B16FO-treated) testis, (C) right (B16FO-treated) testis, x40, showing focal area of melanoma invasion, (D) right (B16FO-treated) testis, x200, showing seminiferous tubules surrounded by melanoma cells.

**Figure 5:**
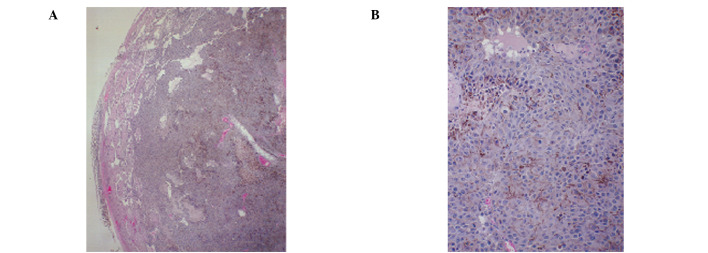
Photomicrographs of B16F0-injected testis in nude mice fed the control diet (4 weeks post-injection) showing invasion of testis by melanoma cells. Magnification: (A) x40, (B) x200.

**Figure 6:**
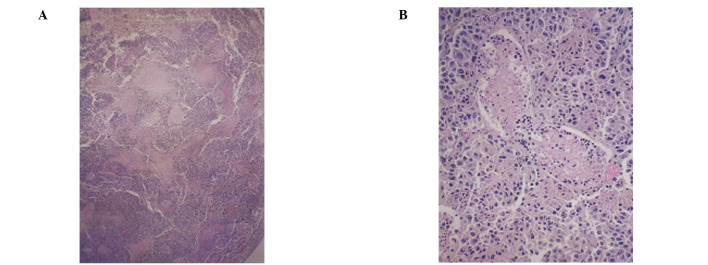
Photomicrographs of B16F0 injected testis in NM 1% diet group of nude mice (4 weeks post-injection) showing melanoma invading testis and degenerating seminiferous tubules. Magnification: (A) x40 and (B) x200.

The right testes in the two groups showed overgrowth of melanoma cells. All of the control mice showed testicular metastasis, while in the NM diet group, only 3 mice showed mild metastasis and 3 mice showed no metastasis. A week after the melanoma injection, the left testis (untreated) of the control mouse was normal, while the right testis showed a focal area of melanoma invasion (Fig. 4). The magnified section (x200) of the right testis (Fig. 4D) shows melanoma cells surrounding the seminiferous tubules at 1 week post-injection. At 4 weeks post-injection, the control mice showed significant testicular invasion by melanoma cells (Fig. 5) in contrast to the less pronounced metastasis in the NM 1% group of mice (Fig. 6).

### Lung metastasis

**Figure 7:**
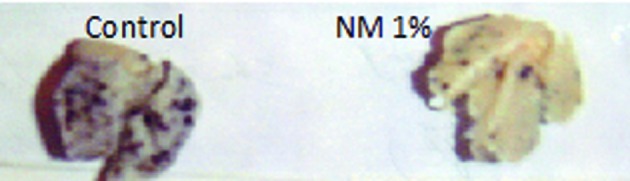
Representative gross images of lungs from nude mice injected with B16FO melanoma cells intratesticularly fed the control (left) or NM 1% diet (right) 4 weeks post injection.

No metastasis to the liver, kidney or spleen was detected in either group. Lung metastasis was observed in 2 of 6 mice in each group. However, severe lung metastasis was observed in the control group, while mild metastasis was detected in the NM 1% group (Fig. 7).

### Mean initial and final weights of mice

No significant difference was found between the initial and final mean weights within the two groups. The mean initial weight of the control group was 37.2±1.3 g and the mean final weight was 38.4±1.6 g. The mean initial weight of the NM 1% group was 36.6±1.5 g and the final weight was 36.7±1.1 g.

## Discussion

The aim of the present study was to investigate the effect of a nutrient mixture on melanoma B16FO growth and metastasis from intratesticular injection into nude mice, representing the lymphatic and hematogenous dissemination of melanoma malignancy. In our study, supplementation with the nutrient mixture suppressed B16FO melanoma cell growth in the testes and metastasis to the peritoneum and lungs after intratesticular injection. All of the mice receiving the control diet exhibited extensive metastasis in the peritoneal cavity, in contrast to the NM 1% diet group, which showed no metastasis in 50% of mice and mild peritoneal metastasis in the remaining mice. No metastasis to liver, kidney or spleen was evident in either of the two groups. Lung metastasis was observed in 2 of 6 mice in each group, with severe lung metastasis being observed in the control group and mild metastasis in the NM 1% group.

Notably, the melanoma cells invaded the peritoneum and metastasized to the lungs from the right testes (injection site of B16FO cells), but did not metastasize to the left testes (untreated), suggesting the testes are not common sites for melanoma B16FO cell metastasis. Previously, we showed that intraperitoneal injection of B16FO melanoma cells into C57BL/6 mice demonstrated intraperitoneal growth and ascites, but did not result in metastasis to other organs (10). In regards to testicular tumor growth, we demonstrated that supplementation with dietary NM significantly suppressed murine melanoma B16FO tumor growth in immune-impaired (athymic) mice. Previous *in vitro* studies have demonstrated significant inhibition of melanoma B16FO and A2058 cell proliferation and strong induction of apoptosis at 500 μg/ml NM, suggesting that inhibition of tumor growth was due probably to induction of apoptosis (12). These findings are in agreement with our *in vivo* findings that exposure of melanoma cells for 18 h to NM before injecting them into mice completely prevented the formation of metastatic lung tumor modules (7).

Degradation of the extracellular matrix (ECM) by matrix metalloproteinases (MMPs) plays a critical role in the formation of tumors and metastasis (13). Findings of studies have shown that highly metastatic melanoma and other cancer cells secrete higher amounts of MMPs as compared to poorly metastatic cells, demonstrating that the invasive and metastatic abilities of these cancer cells correlate with MMP expression, particularly MMP-9 and -2 (14–18). Type IV collagenases MMP-2 and -9 have been the focus of research as type IV collagen is a major structural protein for ECM and basement membrane, and MMP-2 and -9 expression is associated with cancer cell invasion and elevated in a variety of malignancies (19,20). Previous *in vitro* studies have shown that NM significantly inhibited melanoma and other cancer cell MMP-2 and -9 secretion and Matrigel invasion (21). In addition, ECM synthesized by normal fibroblasts treated with NM exhibited increased stability and significantly reduced the osteosarcoma cell growth rate, invasive activity (MMP-2 and -9 secretion and Matrigel invasion) and adhesion to collagen I and other substrates, suppressing tumor growth independently of the immune system function and inhibiting critical steps in cancer metastasis (22).

Rath and Pauling (23) suggested the use of nutritional components, such as vitamin C and lysine and lysine analogues to target plasmin-mediated connective tissue degradation as a universal approach to controlling common pathomechanisms in cancer progression. Lysine interferes with the activation of plasminogen into plasmin by tissue plasminogen activator (tPA) by binding to plasminogen active sites, thereby affecting the plasmin-induced MMP activation cascade (23). Subsequent studies have confirmed this approach and resulted in identifying a novel formulation (NM) comprising lysine, ascorbic acid, proline and green tea extract, and other micronutrients that have shown significant anticancer activity against a large number (∼40) of cancer cell lines, blocking cancer growth, tissue invasion and MMP expression both *in vitro* and *in vivo* (9).

NM is a mixture of nutrients that addresses critical physiological targets in cancer progression and metastasis, such as ECM integrity and MMP activity. Optimal ECM formation and structure is dependent upon adequate supplies of ascorbic acid and the amino acids lysine and proline, which ensure proper synthesis and hydroxylation of collagen fibers. Manganese and copper are also essential for collagen formation. Lysine, a natural inhibitor of plasmin-induced proteolysis, is crucial in supporting ECM stability (23,24). Green tea extract has been shown to be a potent agent in controlling cancer cell growth, metastasis, angiogenesis, and other aspects of cancer progression (25–29). N-acetyl cysteine has been observed to inhibit MMP-9 activity (30) and invasive activities of tumor cells (31). Selenium inhibits MMP secretion and tumor invasion (32), as well as migration of endothelial cells through ECM (31). In addition to addressing ECM properties, some nutrients are critical in inducing cancer cell death. Findings of a previous study confirmed that ascorbic acid inhibits cell division and growth through the production of hydrogen peroxide (33). Since arginine is a precursor of nitric oxide (NO), any deficiency of arginine is capable of limiting the production of NO, which has been shown to predominantly act as an inducer of apoptosis, as in breast cancer cells (34).

In conclusion, the results of the present study have shown that the nutrient mixture was effective in significantly reducing melanoma B16FO cell testicular tumor growth and peritoneal and lung metastasis in male nude mice injected with melanoma cells intratesticularly. These findings together with our earlier results clearly indicate the anticancer potential of NM. Furthermore, use of the nutrient mixture is not likely to pose any toxic effect clinically, especially in the relevant doses, as demonstrated by *in vivo* safety studies. During an *in vivo* study on possible toxicity from NM, we found that NM did not have any adverse effect on vital organs, such as the heart, liver and kidney, nor on the associated functional serum enzymes (35).

## References

[1] Cancer.org Testicular cancer: What are the key statistics about testicular cancer?. http://www.cancer.org/Cancer/TesticularCancer/DetailedGuide/testicular-cancer-key-statistics.

[2] Kinkade S (1999). Testicular Cancer. Am Fam Physician.

[3] Nichols C Testicular Cancer. Testicular Cancer Resource Center.

[4] Hart IR, Fidler IJ (1980). Role of organ selectivity in the determination of metastatic patterns of B16 melanoma. Cancer Res.

[5] Zeidman I, Busso JM (1952). Transpulmonary passage of tumor cell emboli. Cancer Res.

[6] Sugarbaker ED (1952). The organ selectivity of experimentally induced metastases in rats. Cancer.

[7] Roomi MW, Ivanov V, Kalinovsky T, Niedzwiecki A, Rath M (2006). Inhibition of pulmonary metastasis of melanoma B16FO cells in C57BL/6 mice by a nutrient mixture consisting of ascorbic acid, lysine, proline, arginine, and green tea extract. Exp Lung Res.

[8] Gheorgheosu D, Dehelean C, Cristea M, Muntean D (2011). Development of the B16 murine melanoma model. Annals of RSCB.

[9] Niedzwiecki A, Roomi MW, Kalinovsky T, Rath M (2010). Micronutrient synergy - a new tool in effective control of metastasis and other key mechanisms of cancer. Cancer Metastasis Rev.

[10] Roomi MW, Kalinovsky T, Roomi NW, Monterrey J, Rath M, Niedzwiecki A (2008). Suppression of growth and hepatic metastasis of murine B16FO melanoma cells by a novel nutrient mixture. Oncol Rep.

[11] Koshida K, Konaka H, Imao T, Egawa M, Mizokami A, Namiki M (2004). Comparison of two in vivo models for prostate cancer: orthotopic and intratesticular inoculation of LNCaP or PC-3 cells. Int J Urol.

[12] Roomi MW, Kalinovsky T, Niedzwiecki A, Rath M, Tanaka Y (2011). Anticancer effects of a micronutrient mixture on melanoma: modulation of metastasis and other critical parameters. Breakthroughs in Melanoma Research.

[13] Fidler IJ (1990). Critical factors in the biology of human cancer metastasis: twenty-eighth G.H.A. Clowers memorial award lecture. Cancer Res.

[14] Fang W, Li H, Kong L, Niu G, Gao Q, Zhou K, Zheng J, Wu B (2003). Role of matrix metalloproteinases (MMPs) in tumor invasion and metastasis: serial studies on MMPs and TIMPs. Beijing Da Xue Xue Bao.

[15] Liotta LA, Tryggvason K, Garbisa A, Hart I, Foltz CM, Shafie S (1980). Metastatic potential correlates with enzymatic degradation of basement membrane collagen. Nature.

[16] Stetler-Stevenson WG (2001). The role of matrix metalloproteinases in tumor invasion, metastasis and angiogenesis. Surg Oncol Clin N Am.

[17] Duffy MJ (1992). The role of proteolytic enzymes in cancer invasion and metastasis. Clin Exp Metastasis.

[18] Stetler-Stevenson WG, Hewitt R, Corcoran M (1996). Matrix metalloproteinases and tumor invasion from correlation and causality to the clinic. Semin Cancer Biol.

[19] Kleiner DL, Stetler-Stevenson WG (1999). Matrix metalloproteinases and metastasis. Cancer Chemother Pharmacol.

[20] Chambers AF, Matrisian LM (1997). Changing views on the role of matrix metalloproteinases in metastasis. J Natl Cancer Inst.

[21] Roomi MW, Monterrey JC, Kalinovsky T, Rath M, Niedzwiecki A (2010). Inhibition of invasion and MMPs by a nutrient mixture in human cancer cell lines: a correlation study. Exp Oncol.

[22] Ivanov V, Ivanova S, Roomi MW, Kalinovsky T, Niedzwiecki A, Rath M (2007). Naturally produced extracellular matrix inhibits growth rate and invasiveness of human osteosarcoma cancer cells. Med Oncol.

[23] Rath M, Pauling L (1992). Plasmin-induced proteolysis and the role of apoprotein(a), lysine and synthetic analogs. J Orthomolecular Med.

[24] Sun Z, Chen YH, Wang P, Zhang J, Gurewich V, Zhang P, Liu JN (2002). The blockage of high-affinity lysine binding sites of plasminogen by EACA significantly inhibits prourokinase-induced plasminogen activation. Biochem Biophys Acta.

[25] Valcic S, Timmermann BN, Alberts DS, Wachter GA, Krutzsch M, Wymer J, Guillen JM (1996). Inhibitory effect of six green tea catechins and caffeine on the growth of four selected human tumor cell lines. Anticancer Drugs.

[26] Mukhtar H, Ahmed N (2000). Tea polyphenols: prevention of cancer and optimizing health. Am J Clin Nutr.

[27] Yang GY, Liao J, Kim K, Yurtow EJ, Yang CS (1998). Inhibition of growth and induction of apoptosis in human cancer cell lines by tea polyphenols. Carcinogenesis.

[28] Taniguchi S, Fujiki H, Kobayashi H, Go H, Miyado K, Sadano H, Shimokawa R (1992). Effect of (−) epigallocatechin gallate, the main constituent of green tea, on lung metastasis with mouse B16 melanoma cell lines. Cancer Lett.

[29] Hare Y (2001). Green Tea: Health Benefits and Applications.

[30] Kawakami S, Kageyama Y, Fujii, Kihara K, Oshima H (2001). Inhibitory effects of N-acetyl cysteine on invasion and MMP 9 production of T24 human bladder cancer cells. Anticancer Res.

[31] Morini M, Cai T, Aluigi MF, Noonan DM, Masiello L, De Flora S, D’Agostini F, Albini A, Fassina G (1999). The role of the thiol N-acetyl cysteine in the prevention of tumor invasion and angiogenesis. Int J Biol Markers.

[32] Yoon SO, Kim MM, Chung AS (2001). Inhibitory effects of selenite on invasion of HT 1080 tumor cells. J Biol Chem.

[33] Maramag C, Menon M, Balaji KC, Reddy PG, Laxmanan S (1997). Effect of vitamin C on prostate cancer cells in vitro: effect on cell number, viability and DNA synthesis. Prostate.

[34] Cooke JP, Dzau VJ (1997). Nitric oxide synthase: Role in the genesis of vascular disease. Annu Rev Med.

[35] Roomi MW, Ivanov V, Netke SP, Niedzwiecki A, Rath M (2003). Serum markers of the liver, heart, and kidney and lipid profile and histopathology in ODS rats treated with nutrient synergy. J AM Coll Nutr.

